# Prevalence of post-traumatic stress disorder symptoms in adult critical care survivors: a systematic review and meta-analysis

**DOI:** 10.1186/s13054-019-2489-3

**Published:** 2019-06-11

**Authors:** Cássia Righy, Regis Goulart Rosa, Rodrigo Teixeira Amancio da Silva, Renata Kochhann, Celina Borges Migliavaca, Caroline Cabral Robinson, Stefania Pigatto Teche, Cassiano Teixeira, Fernando Augusto Bozza, Maicon Falavigna

**Affiliations:** 1 Instituto Nacional de Infectologia Evandro Chagas, Fundação Oswaldo Cruz, Av. Brasil, 4365, Manguinhos, Rio de Janeiro, RJ 21040-360 Brazil; 2 Instituto Estadual do Cérebro Paulo Niemeyer, Rua do Rezende, 156, Centro, Rio de Janeiro, RJ 20230-026 Brazil; 3 Intensive Care Unit, Hospital Moinhos de Vento (HMV), Rua Ramiro Barcelos, 910, 3° andar, Porto Alegre, RS 90035-001 Brazil; 4 Research Projects Office, HMV, Porto Rua Ramiro Barcelos, 910, 3° andar, Porto Alegre, RS 90035-001 Brazil; 5 Hospital Federal dos Servidores do Estado, Rua Sacadura Cabral, 178, Saúde, Rio de Janeiro, RJ 20221-903 Brazil; 6 National Institute for Health Technology Assessment, Universidade Federal do Rio Grande do Sul, Av. Paulo Gama, 110, Farroupilha, Porto Alegre, RS 90040-060 Brazil; 7 Post-Graduate Program in Psychiatry and Behavioral Sciences, Universidade Federal do Rio Grande do Sul, Porto Alegre, Brazil; 8 PTSD Outpatient program (NET-Trauma), Hospital de Clínicas de Porto Alegre, Porto Alegre, Brazil; 9 D’Or Institute for Research and Education, Rua Diniz Cordeiro, 30, Botafogo, Rio de Janeiro, RJ 22281-100 Brazil

**Keywords:** Critical care, Intensive care units, Meta-analysis, Post-traumatic stress disorder, Prevalence, Systematic review

## Abstract

**Background:**

As more patients are surviving intensive care, mental health concerns in survivors have become a research priority. Among these, post-traumatic stress disorder (PTSD) can have an important impact on the quality of life of critical care survivors. However, data on its burden are conflicting. Therefore, this systematic review and meta-analysis aimed to evaluate the prevalence of PTSD symptoms in adult critical care patients after intensive care unit (ICU) discharge.

**Methods:**

We searched MEDLINE, EMBASE, LILACS, Web of Science, PsycNET, and Scopus databases from inception to September 2018. We included observational studies assessing the prevalence of PTSD symptoms in adult critical care survivors. Two reviewers independently screened studies and extracted data. Studies were meta-analyzed using a random-effects model to estimate PTSD symptom prevalence at different time points, also estimating confidence and prediction intervals. Subgroup and meta-regression analyses were performed to explore heterogeneity. Risk of bias was assessed using the Joanna Briggs Institute tool and the GRADE approach.

**Results:**

Of 13,267 studies retrieved, 48 were included in this review. Overall prevalence of PTSD symptoms was 19.83% (95% confidence interval [CI], 16.72–23.13; *I*^2^ = 90%, low quality of evidence). Prevalence varied widely across studies, with a wide range of expected prevalence (from 3.70 to 43.73% in 95% of settings). Point prevalence estimates were 15.93% (95% CI, 11.15–21.35; *I*^2^ = 90%; 17 studies), 16.80% (95% CI, 13.74–20.09; *I*^2^ = 66%; 13 studies), 18.96% (95% CI, 14.28–24.12; *I*^2^ = 92%; 13 studies), and 20.21% (95% CI, 13.79–27.44; *I*^2^ = 58%; 7 studies) at 3, 6, 12, and > 12 months after discharge, respectively.

**Conclusion:**

PTSD symptoms may affect 1 in every 5 adult critical care survivors, with a high expected prevalence 12 months after discharge. ICU survivors should be screened for PTSD symptoms and cared for accordingly, given the potential negative impact of PTSD on quality of life. In addition, action should be taken to further explore the causal relationship between ICU stay and PTSD, as well as to propose early measures to prevent PTSD in this population.

**Trial registration:**

PROSPERO, CRD42017075124, Registered 6 December 2017.

**Electronic supplementary material:**

The online version of this article (10.1186/s13054-019-2489-3) contains supplementary material, which is available to authorized users.

## Background

Mortality in critical care has steadily declined in recent decades [1, 2]. As a result, concerns about long-term outcomes and quality of life in critical care survivors have become a priority. Recently, more attention has been given to the psychiatric consequences of acute illness in the intensive care unit (ICU), especially in young patients. Psychiatric disorders, such as anxiety, depression, and post-traumatic stress disorder (PTSD), are known to have a strong impact on the quality of life in long-term ICU survivors [3].

PTSD is characterized by having been exposed to an event that is life-threatening or perceived as life-threatening and, subsequently, developing intrusive recollections of the event, hyperarousal symptoms, and avoidant behavior related to the traumatic event [4]. Negative changes in cognition and mood are often part of the clinical picture of PTSD. The classical notion of PTSD as a reaction to warfare or natural disasters has been recently extended to include reaction to road traffic accidents, sexual assaults, and medical conditions such as critical care admission [5]. However, the burden of PTSD associated with critical illness remains unclear.

An in-depth understanding of the current prevalence, risk factors, and accuracy of diagnostic tools is essential to establish early interventions aiming to prevent or minimize PTSD after ICU admission [6]. Prevalence estimates of PTSD among ICU survivors have ranged widely from 4 to 62% [7]. This variability seems to be dependent on the time of PTSD assessment, instrument used, and population studied [7].

Although previous systematic reviews of PTSD prevalence among ICU survivors have been published, there has been increasing interest in this topic in the last few years, and the literature on PTSD in survivors of critical illness has expanded substantially. Moreover, there has been an improvement in methods used for pooling prevalence estimates and interpreting their results. Therefore, given the absence of recent reviews on this topic, we designed the present systematic review and meta-analysis to estimate the overall prevalence of PTSD in adult survivors of critical care.

## Methods

This systematic review and meta-analysis was conducted following the recommendations of the Joanna Briggs Institute (JBI) Reviewers’ Manual [8] and the Preferred Reporting Items for Systematic Reviews and Meta-analyses (PRISMA) Statement [9, 10]. The systematic review protocol was registered with the International Prospective Register of Systematic Reviews (PROSPERO; registration number CRD42017075124).

### Eligibility criteria

The inclusion criteria were defined based on the Condition, Context, Population (CoCoPop) framework, as follows: (1) observational studies (cohort, case-control, cross-sectional studies, or case series) published as full-text articles, (2) context—patients who survived critical care admission, (3) condition—prevalence of PTSD symptoms after ICU discharge, and (4) population analyzed—adult critical care survivors (age ≥ 18 years). We excluded studies that did not report sufficient data to estimate PTSD prevalence, review articles, letters to the editor or comments, studies evaluating neonatal/pediatric critical care units, and studies evaluating patients admitted for acute neurological diseases.

### Data sources and search strategy

We searched the MEDLINE (via PubMed), EMBASE, LILACS, Web of Science, PsycNET, and Scopus databases from inception to September 2018. In addition, we reviewed the reference lists of previous systematic reviews covering the same research question [7, 11, 12]. No language restrictions were imposed. The following search terms were used for all databases: critical care, intensive care units, critical illness, sepsis, and adult respiratory distress syndrome, which were cross-referenced to the terms outcome, follow-up, and post-traumatic stress disorder. The complete search strategies used for all databases are presented in Additional file 1: Table S1.

### Study selection

Two reviewers (CR and RTAS) independently screened titles and abstracts identified by the initial search. The full text of potentially relevant articles was obtained to determine whether the studies met the inclusion criteria. Furthermore, the reference lists of the selected articles were hand-searched to detect any additional studies that had not been identified by the initial electronic search. Disagreements between the two reviewers were resolved by consensus or by involving a third reviewer (FAB) for arbitration.

### Data extraction

Two reviewers (CR and RTAS) independently extracted data from the selected articles, recording the following information if available: (1) study characteristics (location, period of enrollment, criteria for enrollment, number of patients enrolled, population characteristics, duration of follow-up), (2) study design, (3) reason for ICU admission, (4) number of patients evaluated/observed, (5) instrument used for PTSD assessment, (6) prevalence of PTSD after ICU discharge, and (7) time elapsed from discharge to assessment. Any discrepancies were resolved by discussion and consensus among the reviewers (CR, RTAS, FAB). If data were not reported, we contacted the corresponding authors by email.

### Outcomes

The main outcome of interest was the prevalence of PTSD in adult survivors of critical care at different time points after ICU discharge. The diagnosis of PTSD was considered according to each individual study definition.

### Assessment of study quality

We assessed the methodological quality of included studies using the JBI critical appraisal checklist for studies reporting prevalence data [13]. This checklist contains 9 questions, which we divided into 3 domains: participants (questions 1, 2, 4, and 9), outcome measurement (6 and 7), and statistics (3, 5, and 8). A study was rated as having high quality when the methods were appropriate in all 3 domains.

We used the GRADE approach to assess the overall quality of evidence [14]. In the absence of a formal procedure for the assessment of certainty in prevalence estimates, we applied the framework developed for incidence estimates in the context of prognostic studies [15].

### Statistical analysis

We pooled the prevalence estimates from included studies using a random-effects meta-analysis model with the DerSimonian and Laird variance estimator. Prevalence estimates were transformed using the Freeman-Tukey double arcsine transformation so that the data followed an approximately normal distribution. Heterogeneity between studies was assessed by Cochran’s *Q* test and *I*^2^ statistic. Since prevalence estimates vary in different settings due to several factors, such as different patient and ICU characteristics, we also estimated prediction intervals to provide a range of expected PTSD prevalence in different settings [16].

Data from the longest follow-up available in each study were used to estimate the overall prevalence. We performed subgroup analyses to assess whether the method used to diagnose PTSD (screening instrument alone or clinical assessment) and the time point of PTSD assessment (< 3, 3, 6, 12, or > 12 months after ICU admission or discharge) influenced our pooled estimate. We also performed a meta-regression analysis to explore the association between PTSD prevalence estimates and two variables: mean participant age and percentage of respondents in each study. We did not perform a meta-regression analysis for time point of PTSD assessment as a covariate, because we did not expect it to have a linear association with PTSD prevalence.

Results are presented in forest plots with 95% confidence intervals (95% CIs) or scatter plots with point estimates and 95% CI. All analyses were performed using R statistical software version 3.4.4 (R Development Core Team, 2008), with package meta version 4.8-1 [17] and package ggplot2 version 2.2.1 [18].

## Results

**Fig. 1:**
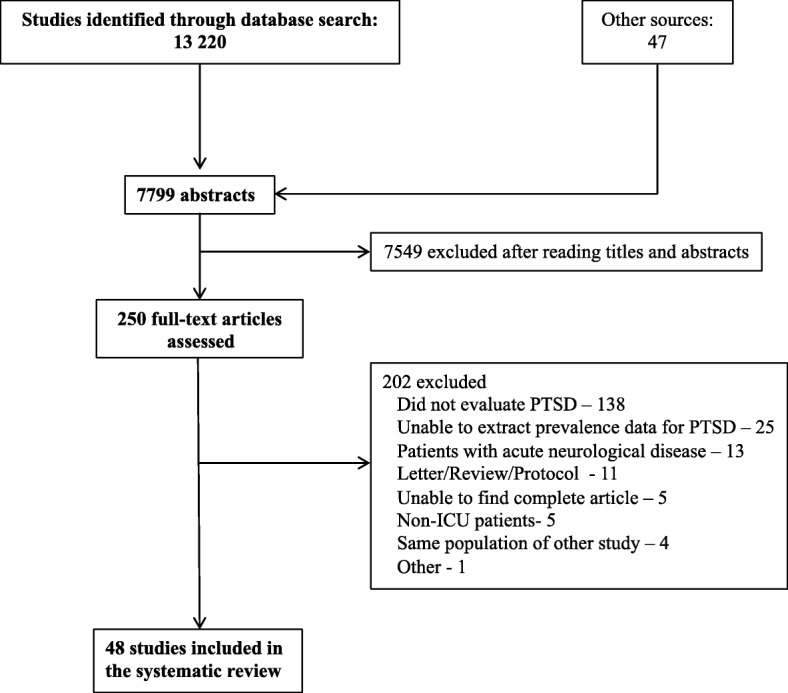
Flowchart of study selection

Of 13,267 records identified, 250 studies were selected for full-text assessment (Fig. 1). Of these, 48 studies enrolling a total of 7152 patients were included in our systematic review and meta-analysis [3, 6, 19–64].

The characteristics of the included studies are shown in Table 1. The time span of the studies was from 1996 to 2018. Most studies were conducted in mixed ICUs (16 studies), followed by medical ICUs (13 studies), trauma ICUs (5 studies), surgical ICUs (3 studies), and long-term and cardiac ICUs (2 studies each). Ten studies did not report the type of ICU involved. The mean age of enrolled patients ranged from 36.5 to 68.0 years; 27 studies reported a male predominance. Except for 4 studies conducted in Australia [20, 25, 33, 62], 2 conducted in Latin America [24, 29], 1 study conducted in Iran [22], and 4 studies in which location was not reported [30, 41, 46, 57], all other studies (77%) were conducted in the USA or Europe.

**Table 1:** Characteristics of included studies

Reference	Study period	Location	Type of ICU	No. of patients	Age, mean ± SD	Male sex, *n* (%)	PTSD prevalence, *n* (%)	Instrument of assessment	Time of assessment
Abraham et al. [19]	Not reported	USA	Trauma ICU	115	42.4 ± 16.7	64 (55.7%)	30 (26%)	DTS	1 year after hospital discharge
Aitken et al. [20]	May 2014–April 2015	Australia	Not reported	57	53.7 ± 14.8	37 (65%)	7 (12.3%)	PCL-5	3–5 months after ICU discharge
Asimakopoulou and Madianos [21]	March 2009–June 2011	Greece	General hospitals	102	45.98 ± 15.17	65 (63.7%)	18 (17.6%)	Mini DSM-IV	3 months after ICU discharge
Bashar et al. [22]	2018	Iran	Mixed ICU	181	65	60 (33%)	181 (100%)	IES-R	3–21 days after ICU discharge
Bienvenu et al. [6]	October 2004–October 2007	USA	Mixed ICU	151 (3 months)	49 ± 14	123 (55%)	36 (23.8%)	IES-R	3, 6, 12, and 24 months after ICU admission
				161 (6 months)			32 (19.8%)		
				141 (12 months)			32 (22.7%)		
				135 (24 months)			32 (23.7%)		
Boer et al. [23]	December 2001–February 2005	Netherlands	Surgical ICU	108	66.8 (57–73)*	41 (38%)	41 (38%)	PTSS-10 and IES-R	1 year after ICU admission
Bugedo et al. [24]	April 2006–January 2007	Chile	Not reported	75	59.5	Not reported	20 (26.66%)	PTSS-10	1 year after ICU admission
Castillo et al. [25]	September 2012–February 2013	Australia	Mixed ICU	101 (3 months)	54 ± 15	98 (70%)	19 (18.8%)	PTSS-10	3 and 6 months after ICU discharge
				92 (6 months)			15 (16.3%)		
Chahraoui et al. [26]	January–June 2013	France	Medical ICU	20	68 ± 8.5	9 (45%)	3 (15%)	IES-R	3 months after ICU discharge
Cox et al. [27]	2009–2010	USA	Mixed ICU	21	56 (47–74)*	9 (43%)	12 (57.1%)	PTSS-10	6 weeks after hospital discharge
Cuthbertson et al. [28]	Not reported	Scotland	Mixed ICU	78	58 (18–87)*	44 (56%)	11 (14.1%)	DSM-IV	3 months after ICU discharge
Da Costa et al. [29]	September 2008–August 2009	Brazil	Medical ICU	138	43.5 (17)	95 (68.8%)	7 (5%)	IES-R	3 months after ICU discharge
Davydow et al. [30]	Not reported	Not reported	Trauma ICU	1456	40.8 (32.0)*	Not reported	364 (25%)	PCL-17	12 months after ICU discharge
Davydow et al. [31]	September 2010–August 2011	USA	Mixed ICU	131 (3 months)	49.0 ± 14.6	69 (57.5%)	20 (15.2%)	PCL-C	3 and 12 months after ICU discharge
				120 (12 months)			18 (15%)		
de Miranda et al. [32]	Not reported	France	Not reported	126	67 (57–75)*	Not reported	26 (20.6%)	IES-R	3 months after ICU discharge
Elliott et al. [33]	Not reported	Australia	Not reported	178	57.20 ± 17.20	116 (65%)	24 (13.5%)	PCL-S	6 months after hospital discharge
Girard et al. [34]	February–May 2001	USA	Medical and cardiac ICU	43	52 (39–65)*	20 (47%)	6 (13.9%)	PTSS-10	6 months after hospital discharge
Granja et al. [35]	January–June 2015	Portugal	Not reported	313	59 (44–71)*	183 (58%)	54 (17.2%)	PTSS-14	6 months after ICU discharge
Griffiths et al. [36]	January 2000–December 2002	England	Not reported	108	56.9	Not reported	56 (54.7%)	PTSS-10	3 months after ICU discharge
Günther et al. [37]	December 2015–March 2016	Sweden	Mixed ICU	30	62 ± 15	18 (60%)	4 (13.3%)	PTSS-10	1 week after ICU discharge
Hauer et al. [38]	Not reported	Germany	Not reported	33	40.3 ± 12.5	16 (48%)	9 (27.3%)	PTSS-10	7.5 ± 2.9 years after ICU discharge
Hauer et al. [39]	July 2004–July 2005	Germany	Cardiac ICU	126	66 ± 9.5	Not reported	15 (11.9%)	PTSS-10	6 months after ICU admission
Hepp et al. [40]	January 1996–June 2000	Sweden	Trauma ICU	90	38.9 ± 13.2	69 (77%)	32 (36%)	CAPS	Up to 3 years after ICU admission
Huang et al. [41]	Not reported	Not reported	Medical ICU	605 (6 months)	49 ± 15	Not reported	148 (24.5%)	IES-R	6 and 12 months after ICU admission
				573 (12 months)			132 (23%)		
Jackson et al. [3]	March 2007–June 2010	USA	Medical or surgical ICU	467 (3 months)	59 (49–69)*	234 (50%)	27 (5.8%)	PCL-S	3 and 12 months after hospital discharge
				467 (12 months)	59 (49–69)*		24 (5.1%)		
Jones et al. [42]	2003–2005	England	Mixed ICU	238	61 (17–86)*	149 (62%)	22 (9.2%)	PTSS-14	3 months after ICU discharge
Jones et al. [43]	2006–2008	Europe	Not reported	332	59.9	210 (63.2%)	29 (8.7%)	TSQ	3 months after ICU discharge
Jónasdóttir et al. [44]	2017	Iceland	Mixed ICU	143	Not reported	M—88 (61.5%)	12/130 (9%) (3 months)	IES-R	3, 6, and 12 months after ICU discharge
							15/110 (14%) (6 months)		
							15/102 (15%) (12 months)		
Jubran et al. [45]	Not reported	USA	Long-term ICU	41	66 (59–72)*	26 (63%)	5 (12.2%)	PTSS-10	3 months after weaning
Kapfhammer et al. [46]	Not reported	Not reported	Not reported	46 (discharge)	36.5 (18.0–50.0)*	Not reported	20 (43.5%)	DSM-IV	At ICU discharge and (average of) 8 years after ICU discharge
Kress et al. [47]	Not reported	USA	Medical ICU	32	48.1	20 (62.5)	6 (18.7)	IES-R	3 months after ICU discharge
Myhren et al. [48]	February 2006–December 2006	Norway	Mixed, medical and cardiac ICU	238	47.9 (15.7)	160 (62.7)	64 (26.8)	IES	4–6 weeks after ICU discharge
Myhren et al. [49]	February 2005–December 2006	Norway	Mixed, medical, and cardiac ICU	180	47.9 (15.7)*	Not reported	48 (26.6%)	IES	12 months after ICU discharge
Nickel et al. [50]	1999–2000	Germany	Medical ICU	41	47.4	Not reported	4 (9.7%)	SCID	3–15 months after ICU discharge (average: 6.2 months)
Richter et al. [51]	Not reported	Germany	Surgical ICU	37	41.7 (17.0)*	28 (76%)	3 (8.1%)	DSM-IV	Mean of 35 (±14) months after ICU discharge
Samuelson et al. [52]	September 2003–March 2005	Sweden	Medical ICU	226	63.3 (13.4)	117 (52%)	19 (8.4%)	IES-R	12 months after ICU discharge
Schellinget al. [53]	Not reported	Germany	Not reported	54	54.2	Not reported	21 (38.8%)	PTSS-10	Not reported
Schelling et al. [54]	Not reported	Germany	Not reported	20	51.8	8 (40%)	8 (40%)	DSM-IV	Median 31 months after ICU discharge
Schnyder et al. [55]	January 1996–June 1997	Switzerland	Trauma ICU	106	37.5 (13.2)	Not reported	5 (4.7%)	DSM-IV	Within 1 month of trauma (median 13.7 days)
Scragg et al. [56]	October 1995–October 1997	England	Medical ICU	80	57.1	42 (52.5%)	12 (15%)	IES	Not reported
Shaw et al. [57]	Not reported	Not reported	Not reported	20	Not reported	Not reported	7 (35%)	IES	Not reported
Strøm et al. [58]	Not reported	Denmark	Mixed, medical and surgical ICU	26	67.0	9 (34.61%)	1 (3.8%)	PTSS-10	2 years after ICU stay
Twigg et al. [59]	December 2000–February 2002	United Kingdom	Medical ICU	44	56.0	20 (45.4%)	10 (22.7%)	PTSS-14	3 months after ICU discharge
Van der Schaaf et al. [60]	June 2004–June 2005	Netherlands	Mixed ICU	255	58.8 (16.6)	166 (69%)	43 (16.8%)	IES	1 year after ICU admission
Wade et al. [61]	November 2008–September 2009	England	Medical ICU	100	57.2 (17.4)	52 (52%)	27 (27%)	PDS	3 months after ICU admission
Wallen et al. [62]	Not reported	Australia	Mixed, medical, surgical and trauma ICU	100	63 (29.8)	68 (68%)	13 (13%)	IES-R	1 month after ICU discharge
Weinert and Sprenkle [63]	2001–2003	USA	Mixed, medical and surgical ICU	80	54.6	Not reported	12 (15%)	PDS	6 months after ICU admission
Wintermann et al. [64]	2017	Germany	Long-term ICU	97	Not reported	73 (75.2%)	29/97 (29.9%)	PTSS-10	3 and 6 months post-transfer (combined result)

*CAPS* Clinician-Administered Post-Traumatic Stress Disorder Scale; *DSM-IV* Diagnostic and Statistical Manual of Mental Disorders, 4th edition; *DTS* Davidson Trauma Scale; *IES* Impact of Event Scale; *IES-R* Impact of Event Scale—revised, *PCL-5* Post-traumatic Stress Disorder Checklist—Civilian V5; *PCL-17* Post-Traumatic Stress Disorder Checklist—Civilian V17; *PCL-C* Post-traumatic Stress Disorder Checklist—Civilian Version; *PCL-S* Post-traumatic Stress Disorder Checklist—Specific Version; *PDS* Posttraumatic Stress Diagnostic Scale; *PTSS-10* Post-Traumatic Stress Syndrome 10-Question Inventory; *PTSS-14* Post-Traumatic Stress Syndrome 14-Question Inventory; *SCID* Structured Clinical Interview; *TSQ* Trauma Screening Questionnaire

*Median (interquartile range)

### Prevalence of PTSD

**Fig. 2:**
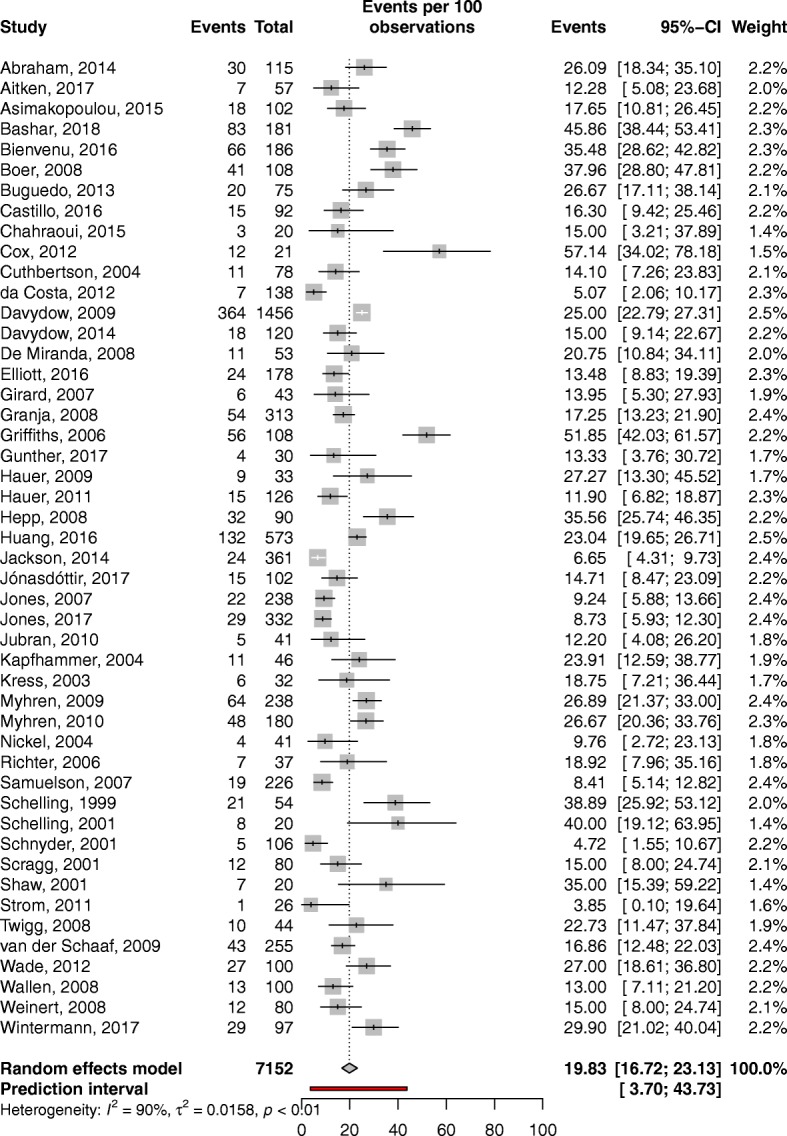
Overall pooled prevalence of post-traumatic stress disorder in adult critical care survivors

The overall pooled prevalence of PTSD symptoms in ICU survivors was 19.83% (95% CI, 16.72–23.13; *I*^2^ = 90%; low quality of evidence) (Fig. 2). The prediction interval for overall PTSD symptoms estimate ranged from 3.70 to 43.73%, with 95% confidence. This prediction interval represents the range of expected PTSD prevalence after ICU discharge in 95% of settings.

**Fig. 3:**
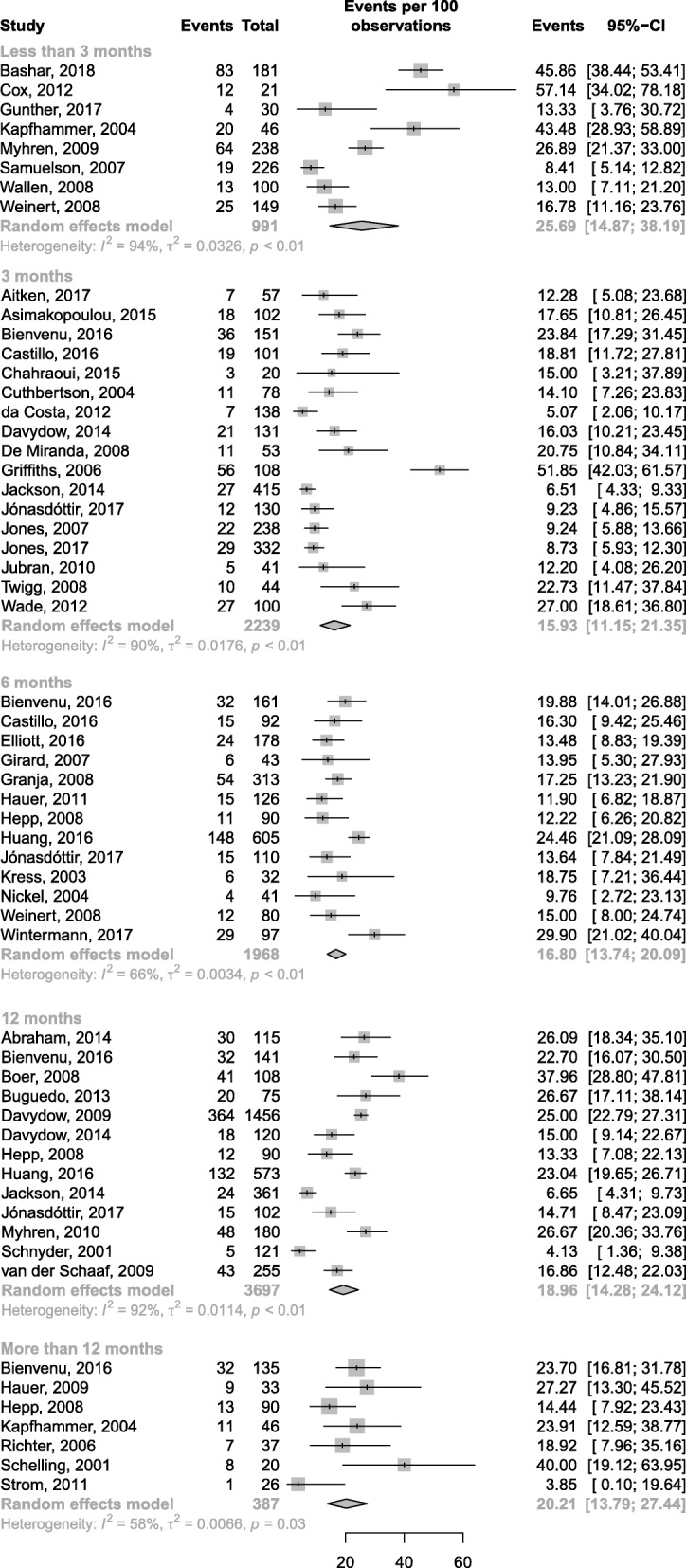
Prevalence of post-traumatic stress disorder according to the time point of assessment

The prevalence of PTSD symptoms ranged from 15.93 to 25.69% according to the time of assessment (Fig. 3). Point prevalence estimates were 15.93% (95% CI, 11.15–21.35.00; *I*^2^ = 90%; 17 studies), 16.80% (95% CI, 13.74–20.09; *I*^2^ = 66%; 13 studies), 18.96% (95% CI, 14.28–24.12; *I*^2^ = 92%; 13 studies), and 20.21% (95% CI, 13.79–27.44; *I*^2^ = 58%; 7 studies) at 3, 6, 12, and > 12 months after discharge, respectively. Eight studies [22, 27, 37, 46, 49, 52, 62, 63] measured the prevalence of symptoms associated with PTSD up to 3 months after ICU discharge, yielding a pooled prevalence estimate of 25.69% (95% CI, 14.87–38.19; *I*^2^ = 94%). However, this high estimate may refer mainly to acute stress disorder rather than PTSD, since in most cases it resolved within 3 months.

**Fig. 4:**
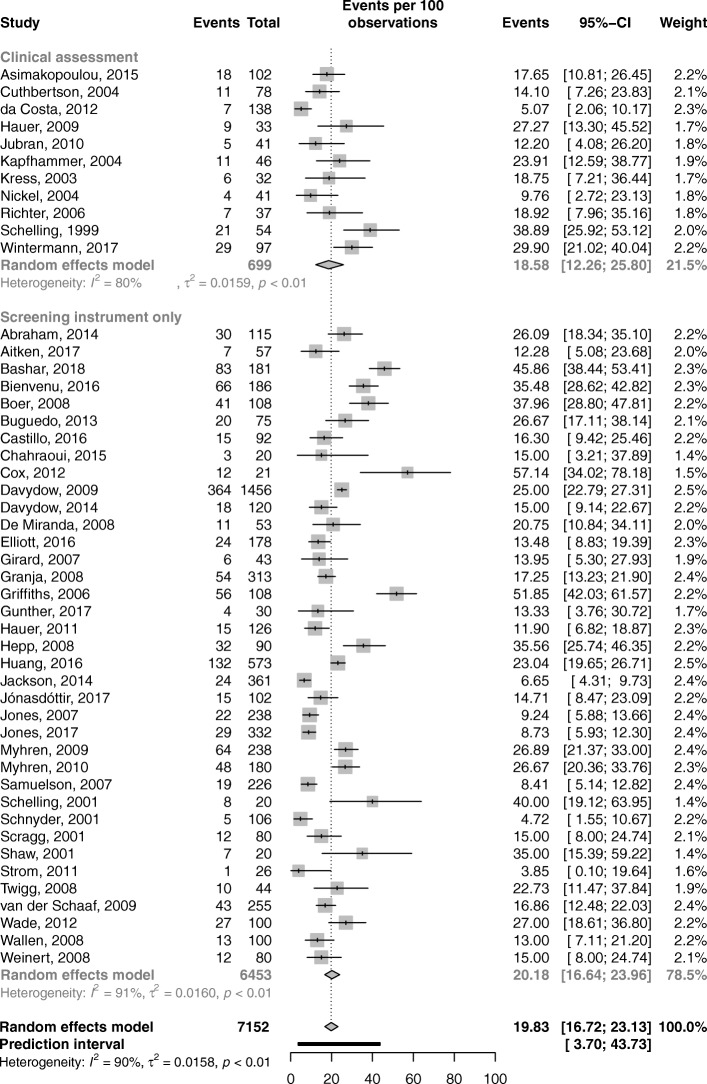
Prevalence of post-traumatic stress disorder according to the assessment method

Subgroup analysis showed that PTSD prevalence as measured by screening instruments alone was 20.18% (95% CI, 16.64–23.96; *I*^2^ = 91%). When the diagnosis was based on clinical assessment, PTSD prevalence was 18.58% (95% CI, 12.26–25.80; *I*^2^ = 80%) (Fig. 4). The difference between these two subgroups was not statistically significant (*p* = 0.71). Additional analyses according to different instruments used at different time points provided similar results (Additional file 1: Table S2, Figure S1, S2, S3, S4, and S5).

Meta-regression analysis showed no linear association between the prevalence of PTSD symptoms and mean participant age or percentage of respondents in the study (data not shown).

### Quality of evidence

A summary of the risk of bias in the included studies, based on the JBI tool, is provided in Additional file 1: Table S3. No study was rated as having high quality; all had limitations in at least 1 of the 3 prespecified domains (participants, outcome measurement, and statistics). Most studies (*n* = 45, 94%) clearly described the study participants and the setting. However, most studies (*n* = 29, 61%) had a study population that did not appropriately address our target population, since they included patients only from specific ICU settings or with specific medical conditions. Twenty-seven studies (56%) did not report how patients were recruited. Eleven studies (23%) had an inadequate response rate. Regarding outcome measurement, most studies (*n* = 45, 94%) assessed PTSD using a standard method for all patients. However, only 10 studies (21%) used clinical assessment to diagnose PTSD, while the other 38 (79%) used only screening instruments. All studies performed appropriate statistical analyses, but the sample size was considered inappropriate in 19 studies (40%).

The overall quality of evidence for PTSD symptoms prevalence estimates was rated as low according to GRADE, mainly because the studies provided only indirect evidence (Additional file 1: Table S4).

## Discussion

In this systematic review and meta-analysis of 48 studies, we found that 1 in every 5 adult survivors of critical care (19.83%) develops PTSD symptoms in the year following ICU discharge. The pooled prevalence of PTSD symptoms in critical care survivors was comparable to that of civilian war survivors (26%) [65], but much higher than that reported in many countries among those exposed to traumatic events (2.5–3.5%) [66]. It was also similar to the 20% prevalence of mental disorder after humanitarian emergencies estimated by the World Health Organization [67]. In the USA, 5.7 million patients are admitted annually to ICUs, with an average mortality rate ranging from 10 to 29% [68]. These data allow us to estimate that approximately 1 million patients develop PTSD after ICU admission annually.

In the present study, the pooled prevalence of PTSD symptoms was 25.69% when measured shortly after ICU discharge (less than 3 months). However, such a high early prevalence of PTSD symptoms may reflect acute stress disorder rather than PTSD. Acute stress symptoms are similar to the post-traumatic stress symptoms that occur within the first month of exposure to a stressor, such as ICU admission [4]. Acute stress disorder may be triggered by fragmented ICU memories of traumatic or psychotic experiences [42] and is a risk factor for the development of PTSD [69]. Although lower, the prevalence range (from 15.93% at 3 months to 18.96% at 12 months) is clinically important, since it may have a negative impact on the quality of life in long-term ICU survivors.

Our systematic review has several limitations. First, despite the use of rigorous, up-to-date methods of data analysis and quality of evidence assessment and a comprehensive search of 6 databases that identified more than 13,000 records, only a few studies reporting data on PTSD prevalence in ICU survivors in specific settings were eligible for inclusion. In addition, most of the included studies had methodological issues that limited the generalizability of the results. Second, PTSD was assessed using different strategies in the included studies. As discussed previously, the diagnosis of PTSD can be challenging, and the use of screening instruments may overestimate PTSD prevalence [70]. However, to date, only a few instruments have been validated for use in the ICU, of which only the Impact of Event Scale—revised [71] and the Post-Traumatic Stress Syndrome 10-Question Inventory have shown good correlation with clinical diagnosis [72]. The lack of proper validation of methods used to evaluate PTSD, as well as their heterogeneity, may have had an impact on the exact prevalence measured in the different studies. However, this impact was minimized in the present systematic review, since similar prevalence estimates of PTSD symptoms were obtained with both clinical assessment (18.58%) and screening instruments (20.18%). Third, there was no parallel assessment of cognitive function in the included studies. An association of long-term PTSD with cognitive dysfunction has been recently reported [73]; however, to date, it remains unknown how cognitive dysfunction can influence PTSD assessment and follow-up, especially regarding consolidation of traumatic memories during mechanical ventilation and sedation. Moreover, PTSD can coexist and be confused with other major psychiatric disorders, such as depression and anxiety [74]. Fourth, the observed statistical heterogeneity was high (90%). However, in contrast with randomized trials, non-controlled studies (e.g., studies of prevalence and incidence) usually have smaller variances and narrower CIs, even with small sample sizes. Thus, a high statistical inconsistency is often expected in meta-analyses of prevalence estimates. Given that the estimates of individual studies included in our meta-analysis ranged mostly from 12 to 30% (similar to the pooled estimate and included in the prediction interval), and we observed consistent results within subgroup analyses (according to instrument used for diagnosis, length of time after ICU stay, and demographic factors), we hypothesize that most of observed inconsistencies may have been the result of the diversity of settings (e.g., patient and ICU characteristics). Fifth, despite the high prevalence observed, it was not possible to establish a direct causal relationship between ICU stay and PTSD, which may be partially explained by other factors, such as the underlying condition of each patient. In this context, action should be taken to further explore the causal relationship between ICU stay and PTSD, as well as to more accurately identify individuals at increased risk of developing PTSD symptoms.

Common stressors in critically ill patients, such as respiratory failure, inflammation, delirium, and communication barriers, may contribute to the occurrence of PTSD, and proper prevention and management of these factors may reduce PTSD incidence after ICU discharge [75]. Also, evidence is emerging that an ICU diary—written by family members or ICU staff—may help patients fill in gaps in their memories, thus reducing the risk of PTSD development [42, 76, 77]. The increased prevalence of PTSD over time in cases that have not received treatment for PTSD symptoms must be highlighted. Although there is little evidence to support the effectiveness of interventions to improve PTSD symptoms among ICU survivors, early treatment with psychotherapy or pharmacological therapy (e.g., antidepressants) may improve quality of life, as observed in PTSD associated with other stressful events [78].

Overall, our findings may have important clinical implications. Despite the high prevalence of PTSD, this disorder is probably underdiagnosed in the post-ICU population. ICU survivors should be screened for PTSD symptoms and cared for accordingly, given the high rates and potential negative impact of PTSD on quality of life. In addition, early and effective measures should be implemented during and after ICU stay to prevent PTSD in this population.

## Conclusion

PTSD symptoms affect a large proportion of critical care survivors, with a high expected prevalence in the first year following discharge from the ICU. Screening of ICU patients for PTSD symptoms, followed by proper support and treatment, is needed, given the potential negative impact of PTSD on quality of life. Additional studies should explore whether a causal relationship exists between ICU stay and PTSD, as well as propose additional measures to prevent and treat PTSD among critically ill patients.

## Additional file

Additional file 1: **Table S1.** Search strategy. **Table S2.** Classification of studies according to the instrument used and the time point of assessment. **Figure S1.** PTSD symptoms assessed with PTSS-10 up to 3 months after an ICU stay. **Figure S2.** Clinical assessment of PTSD and assessment of PTSD symptoms with IES-R, 3 months after an ICU stay. **Figure S3.** PTSD symptoms assessed with IES-R and PTSS-10, 6 months after an ICU stay. **Figure S4.** PTSD symptoms assessed with IES-R and IES, 1 year after an ICU stay. **Figure S5.** Clinical assessment of PTSD assessed more than 1 year after an ICU stay. **Table S3.** Risk of bias in included studies (Joanna Briggs Institute critical appraisal checklist). **Table S4.** Quality of evidence for post-traumatic stress disorder (PTSD) prevalence by the GRADE approach. (DOCX 711 kb)

## Data Availability

All data related to the present systematic review and meta-analysis are available from the corresponding author on reasonable request.

## Abbreviations

CI: Confidence interval
CoCoPop: Condition, Context, Population
ICU: Intensive care unit
JBI: Joanna Briggs Institute
PRISMA: Preferred Reporting Items for Systematic Reviews and Meta-analyses
PROSPERO: International Prospective Register of Systematic Reviews
PTSD: Post-traumatic stress disorder
