# Supplementary material for: Prevalence of post-traumatic stress disorder symptoms in adult critical care survivors: a systematic review and meta-analysis
Source: Crit Care. 2019 Jun 11;23:213. doi: 10.1186/s13054-019-2489-3 (PMC6560853; doi:10.1186/s13054-019-2489-3)
Supplement: Supplementary file 1 — Table S1. Search strategy. Table S2. Classification of studies according to the instrument used and the time point of assessment. Figure S1. PTSD symptoms assessed with PTSS-10 up to 3 months after an ICU stay. Figure S2. Clinical assessment of PTSD and assessment of PTSD symptoms with IES-R, 3 months after an ICU stay. Figure S3. PTSD symptoms assessed with IES-R and PTSS-10, 6 months after an ICU stay. Figure S4. PTSD symptoms assessed with IES-R and IES, 1 year after an ICU stay. Figure S5. Clinical assessment of PTSD assessed more than 1 year after an ICU stay. Table S3. Risk of bias in included studies (Joanna Briggs Institute critical appraisal checklist). Table S4. Quality of evidence for post-traumatic stress disorder (PTSD) prevalence by the GRADE approach. (DOCX 711 kb) [file 13054_2019_2489_MOESM1_ESM.docx]

**ADDITIONAL FILE**

**Prevalence of post-traumatic stress disorder symptoms in adult critical care survivors: a systematic review and meta-analysis**

**Table of contents**

| **Supplementary material** | **Page** |
| --- | --- |
| Table S1. Search strategy | 2 |
| Table S2. Classification of studies according to the instrument used and the moment of assessment | 5 |
| Figure S1. PTSD symptoms assessed with PTSS-10 up to three months after ICU stay. | 7 |
| Figure S2. Clinical assessment of PTSD and assessment of PTSD symptoms with IES-R, three months after ICU stay | 8 |
| Figure S3. PTSD symptoms assessed with IES-R and PTSS-10, six months after ICU stay | 9 |
| Figure S4. PTSD symptoms assessed with IES-R and IES, one year after ICU stay | 10 |
| Figure S5. Clinical assessment of PTSD assessed more than one year after ICU stay | 11 |
| Table S3. Risk of bias in included studies (Joanna-Briggs Institute’s critical appraisal checklist) | 12 |
| Table S4. Quality of evidence for post-traumatic stress disorder (PTSD) prevalence by the GRADE approach | 14 |

**Table S1.** Search strategy.

| MEDLINE (PubMed) | 1. ("Critical Care"[MeSH] OR "Intensive Care Units"[MeSH] OR "critical illness"[MeSH] OR ICU OR Sepsis OR "Respiratory Distress Syndrome, Adult"[MeSH] OR ARDS)  2. ("Outcome Assessment (Health Care)"[MeSH] OR "Patient Outcome Assessment"[MeSH] OR "Follow-Up Studies"[MeSH] OR "Health Status"[MeSH] OR "Morbidity"[MeSH] OR "complications" [Subheading] OR "Long-Term Care"[MeSH] OR "Survivors"[MeSH])  3. (("Anxiety" OR "Anxiety"[MeSH] OR "Depression"[MeSH] OR Depres* OR "Depression"[MeSH] OR "Depressive Disorder"[MeSH]) OR "Stress Disorders, Post-Traumatic"[MeSH] OR "Stress Disorders, Traumatic, Acute"[MeSH] OR Post-Trauma* OR "Cognition"[MeSH] OR “Neurocognitive Disorders"[MeSH] OR "Cognition Disorders"[MeSH] OR "Cognitive Dysfunction"[MeSH] OR (Cogniti* AND (loss OR impairment OR disorder OR sequel OR dysfunction OR decline)) OR "Neuropsychiatry"[MeSH])  4. 1 AND 2 AND 3 |
| --- | --- |
| WEB OF SCIENCE | 1. TS=("Critical Care") OR TS=("Intensive Care Units") OR TS=("critical illness") OR TS=(ICU) OR TS=(Sepsis) OR TS=("Respiratory Distress Syndrome, Adult") OR TS=(ARDS)  2. TS=("Critical Care") OR TS=("Intensive Care Units") OR TS=("critical illness") OR TS=(ICU) OR TS=(Sepsis) OR TS=("Respiratory Distress Syndrome, Adult") OR TS=(ARDS) AND TS=("Outcome Assessment (Health Care)") OR TS=("Patient Outcome Assessment") OR TS=("Follow-Up Studies") OR TS=("Health Status") OR TS=("Morbidity") OR TS=("complications") OR TS=("Long-Term Care") OR TS=("Survivors")  3. TS=("Anxiety") OR TS=("Depression") OR TS=(Depres*) OR TS=("Depression") OR TS=("Depressive Disorder") OR TS=("Stress Disorders, Post-Traumatic") OR TS=("Stress Disorders, Traumatic, Acute") OR TS=("Post-Trauma*") OR TS=("Cognition") OR TS=("Neurocognitive Disorders") OR TS=("Cognition Disorders") OR TS=("Cognitive Dysfunction") OR (TS=(Cogniti*) AND (TS=(loss) OR TS=( impairment) OR TS=(disorder) OR TS=(sequel) OR TS=(dysfunction) OR TS=(decline))) OR TS=("Neuropsychiatry")  4. 1 AND 2 AND 3 |
| SCOPUS | **(**TITLE-ABS-KEY("Critical Care") OR TITLE-ABS-KEY("Intensive Care Units") OR TITLE-ABS-KEY("critical illness") OR TITLE-ABS-KEY(ICU) OR TITLE-ABS-KEY(Sepsis) OR TITLE-ABS-KEY("Respiratory Distress Syndrome, Adult") OR TITLE-ABS-KEY(ARDS)) AND (TITLE-ABS-KEY("Outcome Assessment (Health Care)") OR TITLE-ABS-KEY("Patient Outcome Assessment") OR TITLE-ABS-KEY("Follow-Up Studies") OR TITLE-ABS-KEY("Health Status") OR TITLE-ABS-KEY("Morbidity") OR TITLE-ABS-KEY("complications") OR TITLE-ABS-KEY("Long-Term Care") OR TITLE-ABS-KEY("Survivors")) AND (TITLE-ABS-KEY(Anxiety) OR TITLE-ABS-KEY("Depression") OR TITLE-ABS-KEY(Depres*) OR TITLE-ABS-KEY("Depression") OR TITLE-ABS-KEY("Depressive Disorder") OR TITLE-ABS-KEY("Stress Disorders, Post-Traumatic") OR TITLE-ABS-KEY("Stress Disorders, Traumatic, Acute") OR TITLE-ABS-KEY(Post-Trauma*) OR TITLE-ABS-KEY("Cognition") OR TITLE-ABS-KEY("Neurocognitive Disorders") OR TITLE-ABS-KEY("Cognition Disorders") OR TITLE-ABS-KEY("Cognitive Dysfunction") OR TITLE-ABS-KEY("Neuropsychiatry") OR (TITLE-ABS-KEY(Cogniti*) AND (TITLE-ABS-KEY(loss) OR TITLE-ABS-KEY(impairment) OR TITLE-ABS-KEY(disorder) OR TITLE-ABS-KEY(sequel) OR TITLE-ABS-KEY(dysfunction) OR TITLE-ABS-KEY(decline)))) |
| LILACS | ("Critical Care" OR "Intensive Care Units" OR "critical illness" OR ICU OR Sepsis OR Sepse OR "Respiratory Distress Syndrome, Adult" OR ARDS OR SARA OR "Terapia Intensiva" OR CTI)  AND  ("Outcome Assessment (Health Care)" OR "Patient Outcome Assessment" OR "Follow-Up Studies" OR "Health Status" OR "Morbidity" OR "complications" OR "Long-Term Care" OR "Survivors" OR Desfecho OR Morbidade OR Complicações)  AND  ("Anxiety" OR Depres* OR "Depressive Disorder" OR "Stress Disorders, Post-Traumatic" OR "Stress Disorders, Traumatic, Acute" OR Post-Trauma* OR "Cognition" OR "Neurocognitive Disorders" OR "Cognition Disorders" OR "Cognitive Dysfunction" OR (Cogniti* AND (loss OR impairment OR disorder OR sequel OR dysfunction OR decline)) OR "Neuropsychiatry" OR Delirium OR "Stress Pós Traumático" OR "Sequela Cognitiva" OR "Déficit Cognitivo") |
| PSYCNET | 1. Any Field: "Critical Care" OR Any Field: "Intensive Care Units" OR Any Field:"critical illness" OR Any Field: ICU OR Any Field: Sepsis OR Any Field: "Respiratory Distress Syndrome, Adult" OR Any Field: ARDS  2. Any Field: "Outcome Assessment (Health Care)" ORAny Field: "Patient Outcome Assessment" OR Any Field: "Follow-Up Studies" OR Any Field:"Health Status" OR Any Field: "Morbidity" OR Any Field: "complications" OR Any Field: "Long-Term Care" OR Any Field: "Survivors"  3. Any Field: "Anxiety" OR Depres* OR "Depressive Disorder" OR "Stress Disorders, Post-Traumatic" OR "Stress Disorders, Traumatic, Acute" OR Post-Trauma* OR "Cognition" OR “Neurocognitive Disorders" OR "Cognition Disorders" OR "Cognitive Dysfunction" OR (Cogniti* AND (loss OR impairment OR disorder OR sequel OR dysfunction OR decline)) OR "Neuropsychiatry"  4. 1 AND 2 AND 3 |
| EMBASE | (('outcome assessment (health care)'/de OR 'patient outcome assessment'/de OR 'follow-up studies'/de OR 'health status'/de OR 'morbidity'/de OR complications OR 'long-term care'/de OR 'survivors'/de) AND ('critical care'/de OR 'intensive care units'/de OR 'critical illness'/de OR icu OR sepsis OR 'respiratory distress syndrome, adult'/de OR ards) AND ('anxiety'/de OR depres* OR 'depressive disorder' OR 'depression'/de OR 'stress disorders, post-traumatic'/de OR 'stress disorders, traumatic, acute'/de OR 'post trauma*' OR 'cognition'/de OR 'neurocognitive disorders'/de OR 'cognition disorders'/de OR 'cognitive dysfunction'/de OR (cogniti* AND (loss OR impairment OR disorder OR sequel OR dysfunction OR decline)) OR 'neuropsychiatry'/de)) AND ('article'/it OR 'article in press'/it OR 'letter'/it OR 'review'/it OR 'short survey'/it) |

**Table S2.** Classification of studies according to the instrument used and the moment of assessment.

|  | **< 3 months** | **3 months** | **6 months** | **12 months** | **> 12 months** |
| --- | --- | --- | --- | --- | --- |
| **CAPS** |  |  | Hepp, 2008 | Hepp, 2008 | Hepp, 2008 |
| **DTS** |  | Cuthbertson, 2004 |  | Abraham, 2014 |  |
| **IES** | Myhren, 2009 |  | Hepp, 2008 | Hepp, 2008 Myhren, 2010 | Hepp, 2008 Van der Schaaf, 2009 |
| **IES-R** | Bashar, 2018  Samuelson, 2007  Wallen, 2008 | Bienvenu, 2016 Chahraoui, 2015  Da Costa, 2012 De Miranda, 2008 Jonasdottir, 2017  Kress, 2003 | Bienvenu, 2016 Huang, 2016 Jonasdottir, 2017 | Bienvenu, 2016 Boer, 2008 Huang, 2016 Jonasdottir, 2017  Samuelson, 2007 | Bienvenu, 2016 |
| **Clinical assessment** | Kapfhammer, 2004  Schyneder, 2001 | Asimakopoulou, 2015 Jubran, 2010 |  | Schelling, 2001 | Hauer, 2009 Kapfhammer, 2004  Richter, 2006 |
| **PCL-17** |  |  |  | Davydow, 2009 |  |
| **PCL-5** |  | Aitken, 2017 |  |  |  |
| **PCL-C** |  | Davydow, 2014 | Elliot, 2016 | Davydow, 2014 |  |
| **PCL-S** |  | Jackson, 2014 |  | Jackson, 2014 |  |
| **PDS** |  | Wade, 2012 | Weinert, 2008 |  |  |
| **PTSS-10** | Cox, 2012 Gunther, 2017 | Castillo, 2016  Griffiths, 2006 | Castillo, 2016 Girard, 2007 Hauer, 2011 Wintermann, 2017 | Boer, 2008  Buguedo, 2013 | Strøm, 2011 |
| **PTSS-14** | Bashar, 2018 | Jones, 2007  Twigg. 2008 | Granja, 2008 |  |  |
| **SCID** |  |  | Nickel, 2004 |  |  |
| **TSQ** |  | Jones, 2012 |  |  |  |

CAPS, clinician-administered post-traumatic stress disorder scale; DTS, Davidson trauma scale; IES, impact event scale; IES-R, impact event scale revised; PCL-17, post-traumatic stress disorder checklist (17-item scale); PCL-5, Post-traumatic stress disorder checklist for DSM-5; PCL-C; post-traumatic stress disorder checklist-civilian version; PCL-S, post-traumatic stress disorder checklist-specific stressful experience version; PDS, Posttraumatic Stress Diagnostic Scale; PTSS-10, post-traumatic symptom scale (10-item scale); PTSS-14, post-traumatic symptom scale (14-item scale); SCID, Structured Clinical Interview; TSQ, Trauma Screening Questionnaire

**Figure S1.** PTSD symptoms assessed with IES-R and PTSS-10 up to three months after ICU stay.


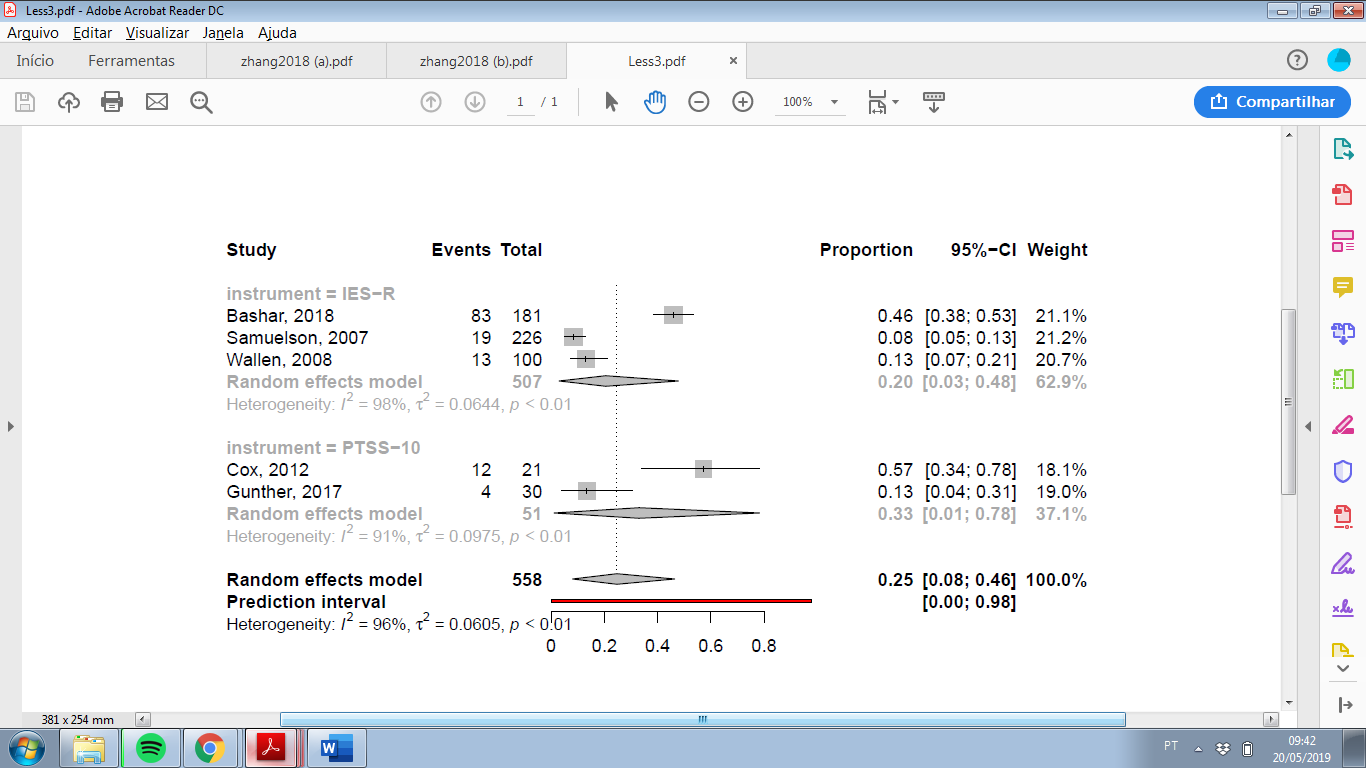


**Figure S2.** Clinical assessment of PTSD and assessment of PTSD symptoms with IES-R, three months after ICU stay.


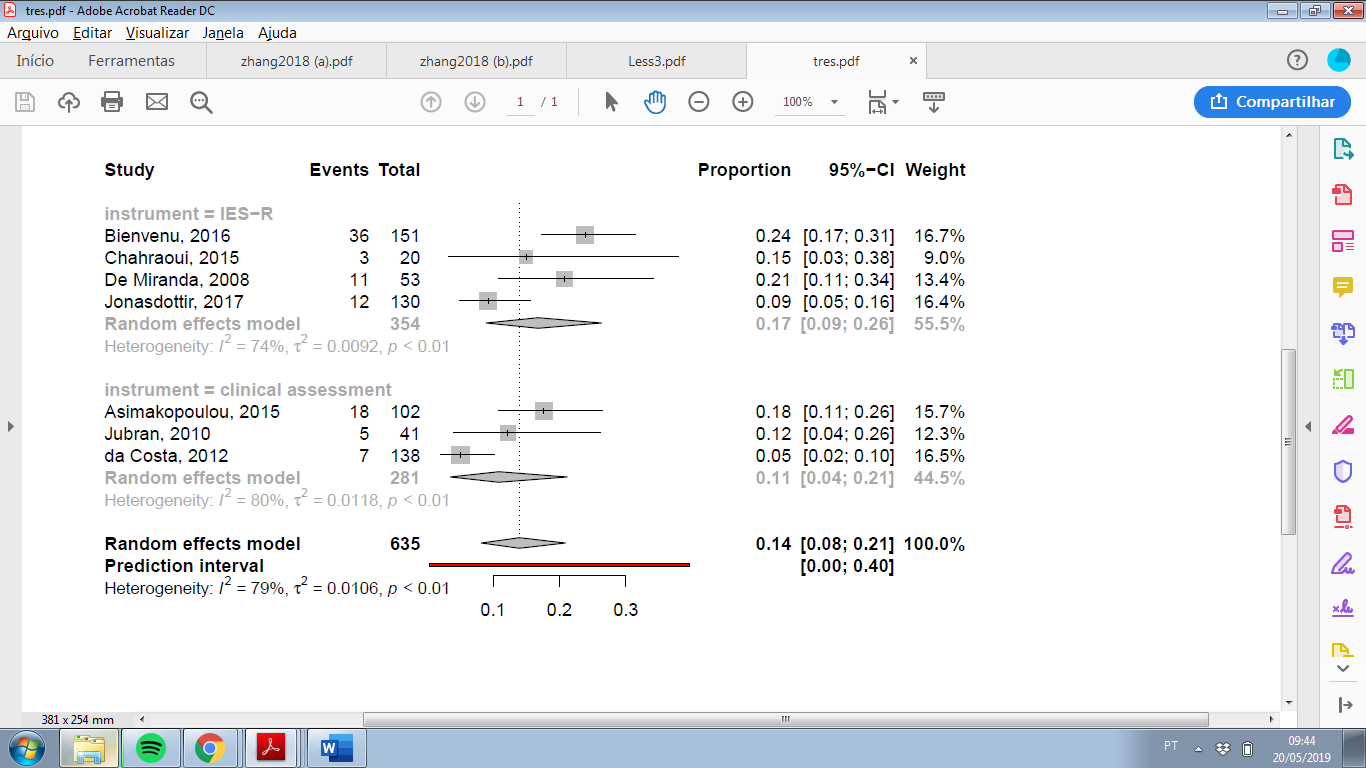


**Figure S3. Clinical assessment of** PTSD and assessement of PTDS symptoms with IES-R and PTSS-10, six months after ICU stay.


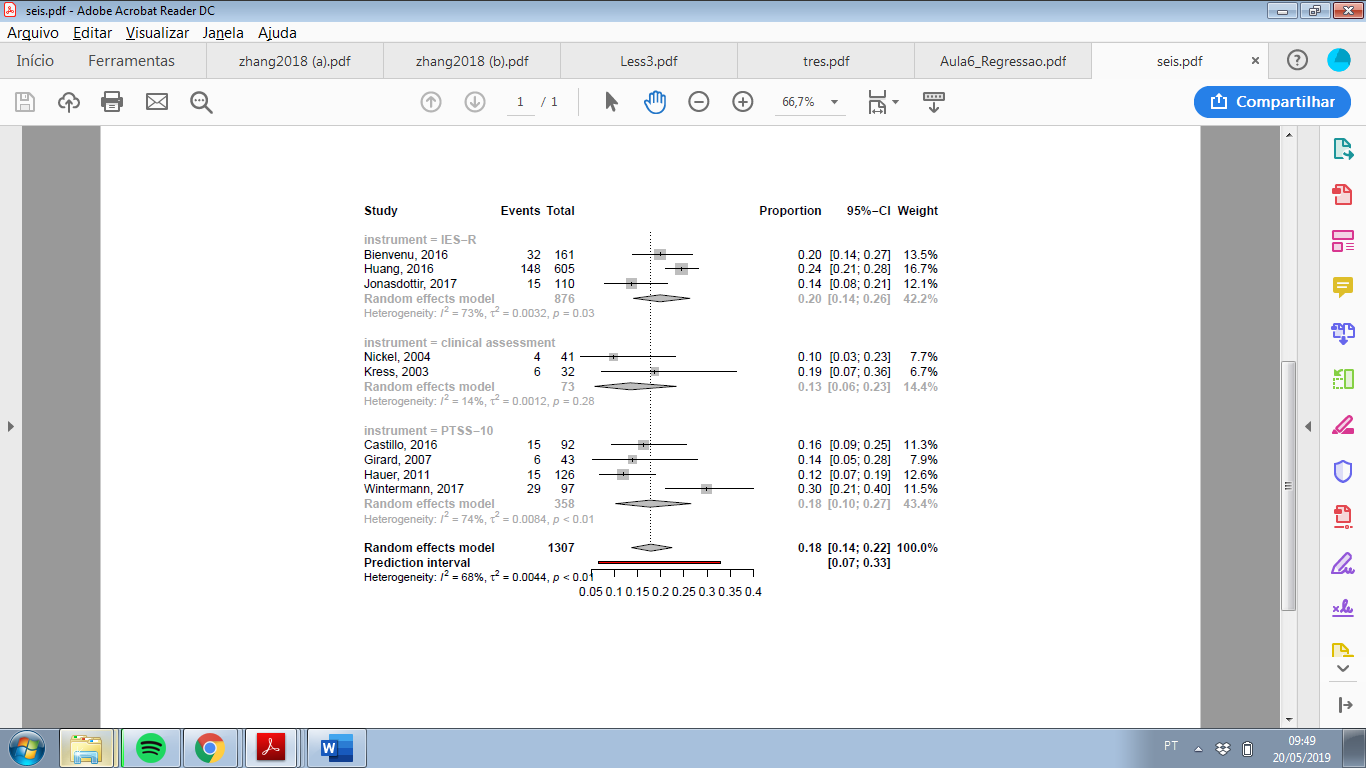


**Figure S4.** PTSD symptoms assessed with IES-R and IES, one year after ICU stay.


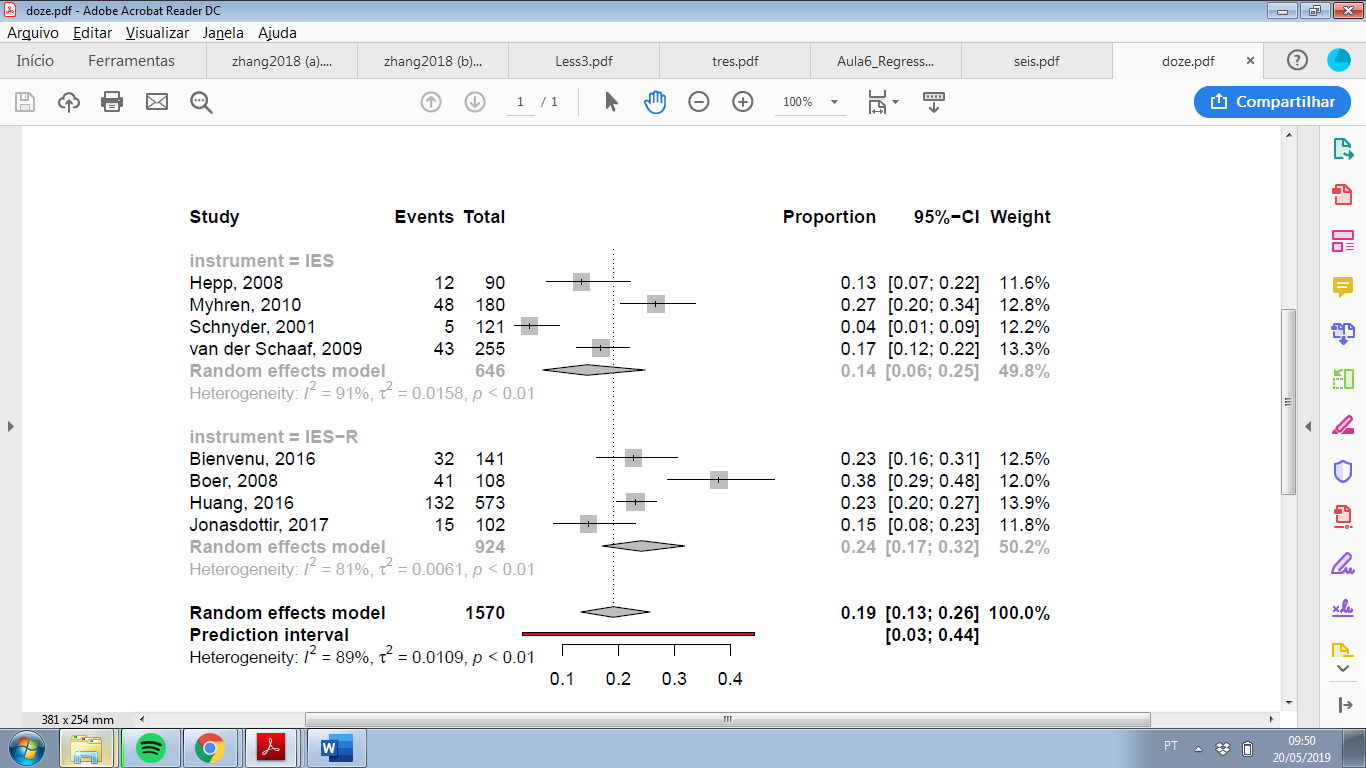


**Figure S5.** Clinical assessment of PTSD assessed more than one year after ICU stay.


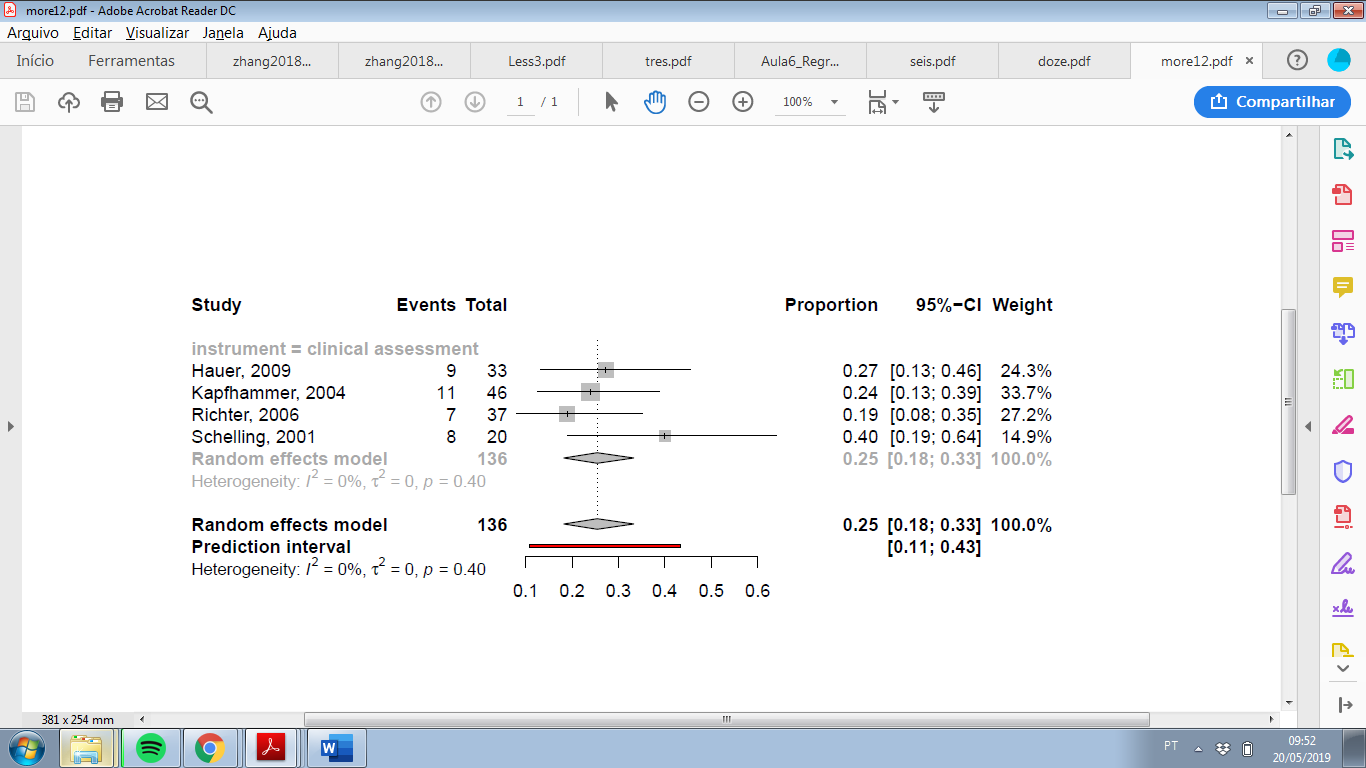


**Table S3.** Risk of bias in included studies (Joanna-Briggs Institute’s critical appraisal checklist).

| **Question** | **Abraham, 2014** | **Aitken, 2017** | **Asimakopoulou, 2015** | **Bashar, 2018** | **Bienvenu, 2016** | **Boer, 2008** | **Buguedo, 2013** | **Castillo, 2016** | **Chahraoui, 2015** | **Cox, 2012** | **Cuthbertson, 2004** | **da Costa, 2012** | **Davydow, 2009** | **Davydow, 2014** | **De Miranda, 2008** | **Elliott, 2016** | **Girard, 2007** | **Granja, 2008** | **Griffiths, 2006** | **Gunther, 2017** | **Hauer, 2009** | **Hauer, 2011** | **Hepp, 2008** | **Huang, 2016** |
| --- | --- | --- | --- | --- | --- | --- | --- | --- | --- | --- | --- | --- | --- | --- | --- | --- | --- | --- | --- | --- | --- | --- | --- | --- |
| 1. Was the sample frame appropriate to address the target population? | N | Y | Y | N | N | N | N | Y | N | Y | Y | Y | N | N | N | Y | N | Y | Y | Y | N | N | N | N |
| 2. Were study participants sampled in an appropriate way? | Y | U | N | Y | Y | U | U | U | Y | Y | Y | Y | U | U | Y | U | U | U | Y | U | U | U | Y | U |
| 3. Was the sample size adequate? | Y | N | Y | Y | Y | Y | N | Y | N | N | N | Y | Y | Y | N | Y | N | Y | Y | N | N | Y | Y | Y |
| 4. Were the study subjects and the setting described in detail? | Y | Y | Y | Y | Y | Y | Y | Y | Y | Y | Y | Y | Y | Y | Y | Y | Y | Y | Y | Y | Y | Y | Y | Y |
| 5. Was the data analysis conducted with sufficient coverage of the identified sample? | Y | N | U | Y | U | Y | U | N | Y | U | Y | U | U | N | U | U | Y | Y | U | U | U | U | Y | U |
| 6. Were valid methods used for the identification of the condition? | N | N | N | N | N | N | N | N | Y | N | N | Y | N | N | N | N | N | N | N | N | Y | N | N | N |
| 7. Was the condition measured in a standard, reliable way for all participants? | Y | Y | Y | Y | N | Y | Y | Y | Y | Y | Y | Y | U | Y | Y | Y | Y | Y | Y | Y | Y | Y | Y | Y |
| 8. Was there appropriate statistical analysis? | Y | Y | Y | Y | Y | Y | Y | Y | Y | Y | Y | Y | Y | Y | Y | Y | Y | Y | Y | Y | Y | Y | Y | Y |
| 9. Was the response rate adequate, and if not, was the low response rate managed appropriately? | Y | Y | Y | Y | Y | Y | N | Y | Y | Y | Y | N | Y | Y | N | Y | N | N | N | Y | Y | Y | Y | Y |
| **Question** | **Jackson, 2014** | **Jónasdóttir, 2017** | **Jones, 2007** | **Jones, 2012** | **Jubran, 2010** | **Kapfhammer, 2004** | **Kress, 2003** | **Myhren, 2009** | **Myhren, 2010** | **Nickel, 2004** | **Richter, 2006** | **Samuelson, 2007** | **Schelling, 1999** | **Schelling, 2001** | **Schnyder, 2001** | **Scragg, 2001** | **Shaw, 2001** | **Strom, 2011** | **Twigg 2008** | **van der Schaaf, 2009** | **Wade, 2012** | **Wallen, 2008** | **Weinert, 2008** | **Wintermann, 2017** |
| 1. Was the sample frame appropriate to address the target population? | N | Y | Y | N | N | N | N | Y | Y | N | N | Y | N | N | N | N | N | N | Y | Y | Y | Y | N | N |
| 2. Were study participants sampled in an appropriate way? | U | U | Y | U | U | U | U | Y | U | Y | U | Y | U | U | Y | Y | U | U | N | Y | Y | U | U | Y |
| 3. Was the sample size adequate? | Y | Y | Y | Y | N | N | N | Y | Y | N | N | Y | N | N | Y | Y | N | N | N | Y | Y | Y | Y | Y |
| 4. Were the study subjects and the setting described in detail? | Y | Y | Y | Y | Y | Y | Y | Y | Y | N | Y | Y | Y | Y | Y | N | N | Y | Y | Y | Y | Y | Y | Y |
| 5. Was the data analysis conducted with sufficient coverage of the identified sample? | N | U | U | Y | Y | Y | U | U | N | N | N | Y | U | Y | Y | U | Y | U | U | N | Y | U | N | N |
| 6. Were valid methods used for the identification of the condition? | N | N | N | N | Y | Y | Y | N | N | Y | Y | N | N | Y | N | N | N | N | N | N | N | N | N | Y |
| 7. Was the condition measured in a standard, reliable way for all participants? | Y | Y | Y | Y | Y | Y | Y | Y | Y | Y | Y | Y | Y | Y | Y | Y | Y | Y | Y | Y | Y | N | Y | Y |
| 8. Was there appropriate statistical analysis? | Y | Y | Y | Y | Y | Y | Y | Y | Y | Y | Y | Y | Y | Y | Y | Y | Y | Y | Y | Y | Y | Y | Y | Y |
| 9. Was the response rate adequate, and if not, was the low response rate managed appropriately? | Y | Y | Y | Y | Y | Y | N | Y | Y | Y | N | Y | Y | Y | Y | Y | Y | N | Y | Y | Y | Y | N | N |

N, No; U, Unclear; Y, Yes. Prespecified domains for quality assessment: a) participants (questions 1, 2, 4, and 9); b) outcome measurement (questions 6 and 7); and c) statistics (questions 3, 5, and 8).

**Table S4.** Quality of evidence for post-traumatic stress disorder (PTSD) prevalence by the GRADE approach.

| **Certainty assessment** | | | | | | | **Summary of findings** |
| --- | --- | --- | --- | --- | --- | --- | --- |
| **No. of participants (studies)** | **Risk of bias** | **Inconsistency** | **Indirectness** | **Imprecision** | **Other** | **Overall certainty of evidence** | **Impact** |
|  |  |  |  |  |  |  |  |
| 5077 (29 studies) | serious^a^ | not serious^b^ | serious^c^ | not serious | none | ⨁⨁◯◯ LOW | The prevalence of PTSD in ICU survivors was 19.83% (95%CI, 16.72-23.13; 95% prediction interval, 3.70-43.73). |

CI, confidence interval; ICU, intensive care unit; PTSD, post-traumatic stress disorder.

Explanations:

a. Most studies did not use appropriate instruments to measure PTSD prevalence and did not report how participants were recruited. Many studies had issues related to sample size and sample coverage.

b. Even though *I*^2^=90%, studies yielded similar results, with the point estimates of most studies ranging from 15 to 25% and showing an overlap of CIs. High I^2^ values are expected in a meta-analysis of prevalence studies, with limited significance in this context. In addition, most probable cause of statistical heterogeneity is the difference in study population, factor that has been penalized in the assessment of indirectness domain.

c. Many studies included patients from specific settings (such as patients with specific medical conditions), rather than from general ICUs, contributing for the sample heterogeneity.
